# On the survival of 48 h *Plasmodium vivax* Aotus monkey-derived ex vivo cultures: the role of leucocytes filtration and chemically defined lipid concentrate media supplementation

**DOI:** 10.1186/s12936-020-03348-9

**Published:** 2020-08-03

**Authors:** Nicanor Obaldía, Marlon Nuñez

**Affiliations:** 1 Center for the Evaluation of Antimalarial Drugs and Vaccines, Tropical Medicine Research/Instituto Conmemorativo Gorgas de Estudios de la Salud, Panama city, Panama; 2 Center for Global Health & Infectious Diseases Research, Department of Global Health, University of South Florida, Tampa, FL USA; 3 Department of Immunology and Infectious Diseases, Harvard, T.H. Chan School of Public Health, Boston, MA USA

**Keywords:** *Plasmodium vivax*, *Ex*-*vivo* cultures, *Aotus*, Non-human primates, Animal model

## Abstract

**Background:**

Filtration of leukocytes (WBCs) is a standard practice of malaria ex vivo cultures. To date, few studies have considered the effect of filtration or the lack thereof on the survival of *Plasmodium vivax* ex vivo cultures through one cycle of maturation. This study investigates the effect of WBC filtration and culture media supplementation on the survival of 48–72 h ex vivo cultures.

**Methods:**

Using parasitaemia density, the study compares the survival of Plasmodipur^®^ filtered, filter-retained or washed ex vivo cultures, maintained with McCoy’s5A medium supplemented with 25% serum alone or 20% in combination with 5% chemically defined lipid concentrate (CDLC), and in washed ex vivo cultures plus GlutaMAX™, benchmarked against IMDM™ or AIM-V™ media; also, assessed the survival of ex vivo cultures co-cultivated with human red blood cells (hRBCs).

**Results:**

After 48 h of incubation a statistically significant difference was detected in the survival proportions of filtered and the filter-retained ex vivo cultures supplemented with serum plus CDLC (*p* = 0.0255), but not with serum alone (*p* = 0.1646). To corroborate these finding, parasitaemias of washed ex vivo cultures maintained with McCoy’s5A complete medium were benchmarked against IMDM™ or AIM-V™ media; again, a statistically significant difference was detected in the cultures supplemented with CDLC and GlutaMAX™ (*p* = 0.03), but not when supplemented with either alone; revealing a pattern of McCoy’s5A medium supplementation for *Aotus*-derived *P. vivax* cultures as follows: serum < serum + GlutaMAX™ < serum + CDLC < serum + CDLC + GlutaMAX™; confirming a key role of CDLC in combination with GlutaMAX™ in the enhanced survival observed. Lastly, results showed that co-cultivation with malaria-naïve hRBCs improved the survival of ex vivo cultures.

**Conclusions:**

This study demonstrates that WBC filtration is not essential for the survival of *P. vivax* ex vivo cultures. It also demonstrates that McCoy’s5A complete medium improves the survival of *Aotus*-derived *P. vivax* ex vivo cultures, with no significant difference in survival compared to IMDM and AIM-V media. Finally, the study demonstrates that co-cultivation with hRBCs enhances the survival of ex vivo cultures. These findings are expected to help optimize seeding material for long-term *P. vivax* in vitro culture.

## Background

Malaria, caused by protozoan parasites transmitted to man by the bites of the female *Anopheles* mosquitoes, every year affects millions of people worldwide [1]. *Plasmodium vivax* threatens approximately 40% of the world’s population [2], producing extensive morbidity and important mortality [3–7] with 7.5 million cases reported in 2018 [8]; 85% of which originated outside of Africa, particularly in Southeast Asia, the Western Pacific and South America [9], with India accounting for 47% of all vivax cases globally [8], and increasing evidence that the infection is spread across sub-Saharan Africa [10].

Even though, the burden of vivax malaria has decreased from about 24.5 million cases in 2000 to 14.3 million cases in 2017, recent evidence suggests that the rates of decline have stalled, and the infection has even increased in some regions [11], raising the possibility that vivax malaria will remain endemic after the elimination of *Plasmodium falciparum* [12]; due in part to its peculiar biology, that includes the early formation of gametocytes before the appearance of symptoms; as well as the presence of dormant liver stages known as hypnozoites [12, 13], and the recently reported bone marrow cryptic infection reservoir [14], both that are believed to be implicated in the recurrences observed in infected individuals [12, 13, 15]. Yet, the lack of a continuous in vitro culture has limited research in its biology, pathophysiology and the development of new drugs, vaccines and diagnostic tools to combat and eliminate this neglected tropical disease [16].

*Plasmodium vivax* has proven difficult to maintain in continuous in vitro culture beyond 48–72 h of incubation, due mainly to its special culture conditions [17], its preference at invading young reticulocytes that are restricted to the bone marrow [18–23] and a plethora of other factors that may impact the fitness of the parasite [24].

Non-human primate *P. vivax*-infected blood has been an important source of parasites for long-term in vitro culture attempts [25–27], but parasitaemias of *Aotus*-derived ex vivo cultures of *P. vivax* are also rarely sustainable beyond 48–72 h of incubation [24, 25, 28]. Hence, a clear understanding of the culture conditions and nutrient media supplementation requirements for the survival of *Aotus*-derived ex vivo cultures during the critical first 48–72 h of incubation is essential for the establishment of a long-term in vitro culture [21].

*Aotus* monkeys have been used as donors of *P. vivax*-infected red blood cells (iRBCs) to initiate in vitro continuous cultures with different degrees of success [26, 29]. So far, different culture conditions and culture media containing variable concentrations of human sera, cholesterol and supplements such as GlutaMAX™ have been tried in ex vivo cultures of *P. vivax* [21, 24, 30–32], however optimal culture conditions and a definitive media supplementation regime for *Aotus*-derived ex vivo cultures of *P. vivax* remain elusive [21].

Studies have demonstrated that *Plasmodium* species are incapable of de novo biosynthesis of fatty acids and cholesterol and must obtain it from the host [33, 34]. It is also known that *Aotus* serum does not support in vitro culture of *P. falciparum* [35–37], possibly to low concentrations of essential nutrients such as cholesterol and lipids, that are known to be in balance both in serum and red blood cells [34]. Recently however, it was established that malaria parasites are in high demand for lipids during replication and enlargement of their parasitophorous vacuole membrane (PVM) in the liver [38], but little is known about the demands for lipids and cholesterol of *P. vivax* parasites during replication in ex vivo culture.

During attempts to adapt field isolates of Panamanian *P. vivax* parasites to splenectomized *Aotus* monkeys, the observation that a tube containing *P. vivax*-infected EDTA anticoagulated whole blood (without leucocyte depletion), left at room temperature for three days was still infectious to *Aotus* monkeys (Obaldia NIII. unpublished observations); prompted the hypothesis that depletion of leukocytes (WBCs) by filtration, as has been customary to avoid phagocytosis or contamination for in vitro studies [39–41], was not essential to initiate short-term *P. vivax* ex vivo cultures; but that leukocytes may contribute to maintain a homeostatic milieu mimicking whole blood conditions ex vivo instead.

Different blood leucodepletion methods have been used to remove unwanted WBCs from *P. vivax*-infected blood for ex vivo *cultures* [28, 39, 40, 42, 43], with some researchers favouring and others arguing against it [21]. Nonetheless, during this process, a large proportion of *P. vivax* iRBCs are retained by the filters or lysed and lost as seeding material to initiate ex vivo cultures. Therefore, with the objective of improving the survival of *P. vivax Aotus*-derived ex vivo cultures, and optimizing seeding material for long-term in vitro cultures, this study assessed the effect of filtration and lipid culture media supplementation or co-cultivation with fresh human red blood cells (hRBCs), over the survival of *P. vivax Aotus*-derived ex vivo cultures through one cycle of maturation.

The findings of this study are expected to further contribute to the optimization of seeding material for the long-term in vitro culture of *Aotus*-derived *P. vivax*.

## Methods

### Malaria parasites

The *Aotus*-adapted *P. vivax* Salvador I (SAL-I) strain originally obtained from William C. Collins (CDC, Atlanta, USA) [44], and further adapted by serial passage to spleen intact Panamanian *Aotus lemurinus lemurinus* monkeys [45] was used for inoculation of the donor monkeys of this study.

### Animals

Twenty-two (22) laboratory-bred, spleen-intact, male and female *Aotus l. lemurinus* (Panamanian Owl monkey), karyotype VIII and IX [46], weighing between 758 and 829 g were used as *P. vivax*-infected blood donors. The animals were housed at the Gorgas Memorial Institute of Health Studies (GMIHS) monkey colony in Panama City, Panama, and cared and maintained as described [47].

### Ethics

The experimental protocol was approved by the GMIHS Institutional Laboratory Animal Care and Use Committee in accordance with procedures described in the *Guide for the Care and Use of Laboratory Animals, 1996*; protocol approval number 2013/02.

### Animal inoculations

Briefly, monkeys were immobilized with ketamine administered intramuscularly in the thigh muscle at 10 mg/kg and inoculated intravenously (iv) in the saphenous vein using a 25-g butterfly needle catheter attached to a 3-ml syringe containing *P. vivax*-infected blood from a donor monkey. Previously, 1 ml of venous blood had been obtained from the femoral vein of the donor animal with a 21-g butterfly needle catheter attached to a 3-ml syringe containing Sodium Citrate Solution (Sigma-Aldrich, St. Louis, MO, USA). Blood was then centrifuged at low speed (1200 rpm) for 5 min and plasma removed and further washed three times with phosphate buffer saline (PBS). The iRBCs pellet was then re-suspended in 1 ml of RPMI incomplete media to contain 5 × 10^6^ iRBCs x ml. The animals were followed up daily for parasitaemia determination from day 5 post-inoculation (PI) until peak parasitaemia that occurred approximately between days 12 and 21 (PI) [45], with daily thick and thin blood smears obtained from a prick in the marginal ear vein using a lancet and stained with Giemsa as described [48]. At peak parasitaemia, 3 ml of blood were obtained from the femoral vein of fasted animals for ex vivo cultures as described above [45, 49], at this point the animals received radical treatment with mefloquine at 20 mg/kg by gastric intubation as described [45].

### Blood preparation

**Fig. 1:**
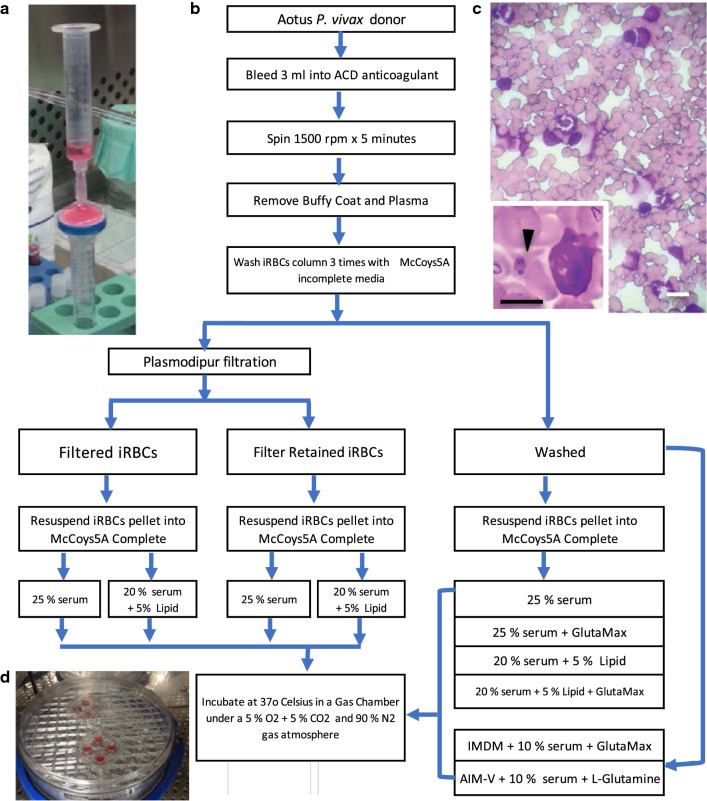
Experimental design flow diagram. **a** Plasmodipur^®^ filter and 10-ml syringe assembly for reverse back flush of filter-retained iRBCs; **b** Experimental design flow diagram; **c** Representative image of filter-retained *P. vivax Aotus* iRBCs containing abundant leukocytes (WBCs). Inset: black arrowhead indicating an early throphozoite (ring) in proximity to a monocyte. Black bar = 5 µm; white bar = 10 µm; **d** Image of incubation gas chamber base showing two 4-well *P. vivax* ex vivo culture plates. iRBCs = infected red blood cells

Briefly, anticoagulated whole blood was centrifuged at 1200 rpm for 5 min, followed by removal of the buffy-coat and plasma with the help of a serological pipette. The remaining iRBCs column was further re-suspended and washed twice by centrifugation as above with incomplete McCoy’s5A medium (Sigma-Aldrich, St. Louis, MO, USA), re-suspended in incomplete McCoy’s5A medium and either used directly (washed) or passed through a Plasmodipur^®^ filter (EuroProxima, Amhem, The Netherlands) to remove remaining WBCs. Filter-retained iRBCs were back flushed from the Plasmodipur^®^ filter previously filled with 3 ml of incomplete McCoy’s media, applying steady suction with the help of a 10-ml syringe, and further washed three times as described above before plating (Fig. 1).

### *Ex*-*vivo* cultures

Plasmodipur^®^-filtered, filter-retained or washed-only (unfiltered) iRBCs, were re-suspended to 5% haematocrit (HCT) in complete McCoy’s5A Modified medium (Sigma-Aldrich, St. Louis, MO, USA) containing 25 mM HEPES (Sigma-Aldrich, St. Louis, MO, USA), 2 g/mL sodium bicarbonate (Sigma-Aldrich, St. Louis, MO, USA), 2 g/L d-glucose (Sigma-Aldrich, St. Louis, MO, USA) and 40 μg/mL gentamycin (Sigma-Aldrich, St. Louis, MO, USA) [25], supplemented with 25% heat inactivated human AB + serum alone or 20% in combination with 5% chemically defined lipid concentrate (CDLC) (Cat No. 11905031, GIBCO, Grand Island, NY, USA) alone or in combination with 200 nM GlutaMax™-I 100X (GIBCO, Life Technologies, CA, USA) (a stabilized form of l-glutamine, L-alanyl-l-glutamine, that prevents degradation and ammonium build-up during long-term cultures); IMDM™ 1x + GlutaMAX™ (Iscove’s modified Dulbecco’s containing + 25 mM HEPES + 3.024 g/L Sodium Bicarbonate) (GIBCO, Life Technologies, CA, USA) culture medium supplemented with 10% heat inactivated human AB + serum as described [32]; and AIM-V 1x (+ l-Glutamine + 50 ug/ml Streptomycin Sulfate + 10 ug/mL Gentamicin Sulfate) (GIBCO, Life Technologies, CA, USA) culture medium supplemented with 10% heat inactivated human AB + serum as described [27]. In general, 25 μL of packed iRBCs (filtered, filter-retained or washed) were re-suspended in a 1.8 ml sterile Eppendorf™ tube containing 475 μL of complete culture media supplemented with either 25% serum alone or 20% in combination with 5% CDLC, previously brought to 37 °C in an incubator to obtain a 5% HCT final cell suspension. Each iRBC cell suspension tube was then seeded into one well of a 4-well round Nunc culture plate (Thermo Scientific, USA) (Fig. 1). For the co-cultivation experiment *Aotus P. vivax* iRBCs were mixed in a 50:50 proportion with malaria-naïve *Aotus* RBCs or hRBCs and then brought to 5% HTC concentration in McCoy’s5A medium supplemented with 25% sera as described above. Plates were incubated at 37 °C in a gas chamber under a 5% O_2_ + 5% CO_2_ and 90% N_2_ gas atmosphere in a Thermo^®^ incubator in static conditions. Culture media was replaced at 24 h.

### Parasitaemia density and developmental stages differentials proportions determination

Parasitaemias were followed up through one cycle of maturation. Thin smears were prepared at 0, 24 and 48 h and in three donor animals up to 72 h of incubation, stained with Giemsa for the determination of  % parasitaemia and parasite stages differential proportions (% parasitaemia = number of iRBCs in 5000 RBCs × 100%). At the end of the 48–72 h of incubation, cultures were harvested, and samples were preserved in Trizol and IFA slides prepared for a parallel experiment.

### *Ex*-*vivo* cultures survival definition

Survival of ex vivo cultures was defined as cultures with any microscopic detectable parasitaemia through one cycle of maturation (48–72 h of incubation).

### Optimization of reference ex vivo culture media serum concentration

In order to determine the optimal serum concentration for the survival of *P. vivax* ex vivo culture in the reference group (McCoy’s5A complete + serum), washed iRBCs obtained from *Aotus* donor 22033 were cultured with McCoy’s5A media supplemented with either 25 or 50% serum alone in triplicate as described above, determining its parasitaemias at 0, 24 and 48 h of incubation.

### Statistical analysis

Plotting and parametric and non-parametric statistical analysis were performed using the Prism 6 for Mac OS x (Graph Pad^®^ Software, San Diego, CA, USA).

## Results

### Filtration of leukocytes is not essential for the survival of *Plasmodium vivax Aotus*-derived ex vivo cultures

The observation that Plasmodipur^®^ filter-retained iRBCS (retained) ex vivo cultures, survived longer than filtered iRBCs during attempts to grow *Aotus*-derived *P. vivax* parasites in vitro, prompted the design of an experiment to assess the effect of filtration on the survival of filtered *versus* retained *P. vivax* SAL-1 *Aotus*-derived iRBCs ex vivo cultures, maintained with McCoy’s5A culture medium supplemented with either 25% serum alone or 20% in combination with 5% CDLC, determining their endpoint parasitaemias at 48 or 72 h of incubation (Fig. 1). Ten *Aotus P. vivax*-infected blood donors, bled at peak parasitaemia (Mean = 101.46 × 10^3^ × µL), were used in the experiment (Additional file 1: Table S1).

First, to determine the optimal serum concentration for culturing *P. vivax* Aotus derived iRBCs, washed iRBCs,obtained from donor *Aotus* monkey MN22033 were maintained with McCoy’s5A medium supplemented with either 25 or 50% serum in three technical replicates for 48 h of incubation as described above. As shown on Additional file 2: Fig. S1, cultures maintained with 25% serum remained viable up to 48 h of incubation while those cultures maintained with media supplemented with 50% serum did not. Hence, 25% serum concentration was chosen for the reference group.

**Fig. 2:**
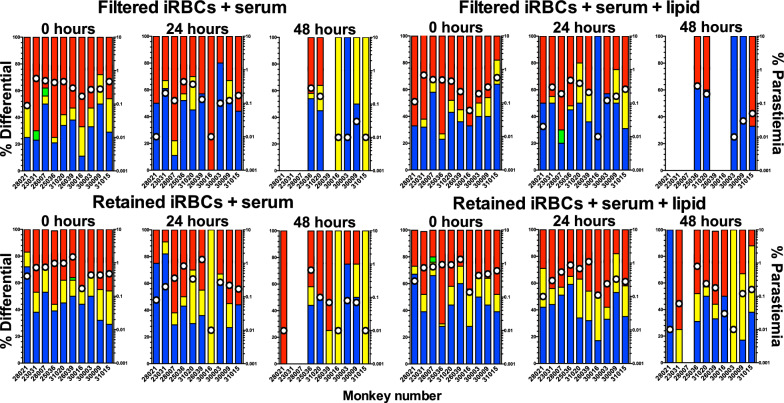
Parasitaemias and developmental stages differential proportions plots of individual *Plasmodium vivax Aotus*-derived ex vivo cultures. Plasmodipur^®^ filtered iRBCs and filter-retained iRBCs cultures were maintained with McCoy’s5A medium supplemented with 25% serum alone or 20% serum in combination with 5% chemically defined lipid concentrate. iRBCs = infected red blood cells. Parasitaemia density = white dots. Bars: red = rings; blue = trophozoites; yellow = schizonts; green = gametocytes

**Fig. 3:**
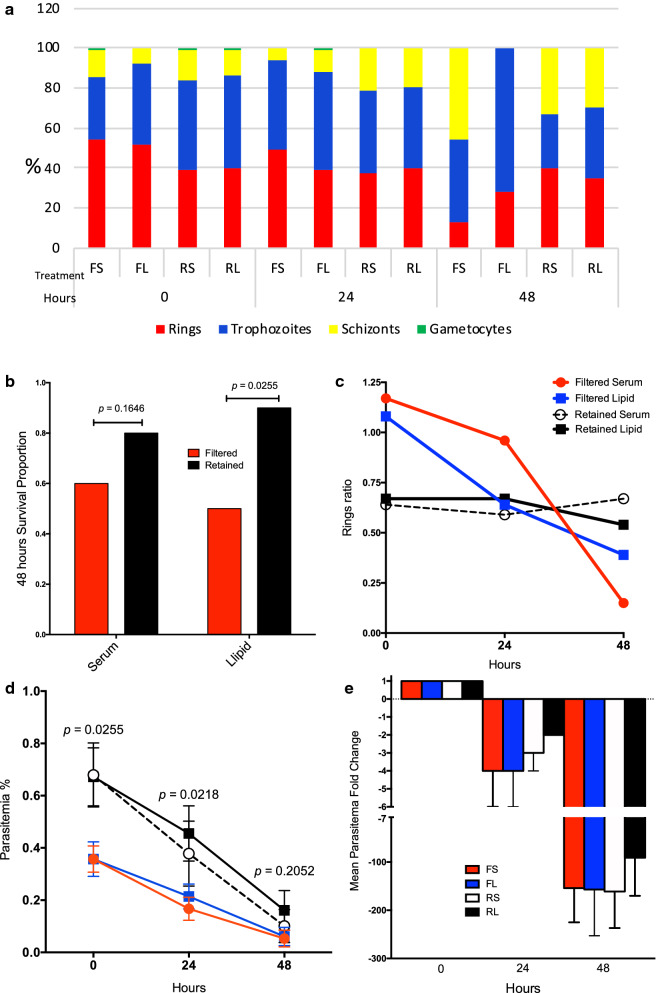
*Plasmodium vivax* Aotus-derived ex vivo cultures. **a** Mean developmental stages proportions of experimental treatment groups. **b** 48 h ex vivo cultures survival proportions (*p *= one-tailed Chi square test). **c** Mean rings ratios of experimental treatment groups. **d** Mean parasitaemia of experimental treatment groups (*p* = Mann–Whitney, two-tailed t-test). **e** Mean parasitaemia fold change of experimental treatment groups. *FS* filtered serum, *FL* filtered lipid, *RS* retained serum, *RL* retained lipid. Cultures were maintained with McCoy’s 5A medium supplemented with 25% serum alone (S) or 20% serum in combination with 5% chemically defined lipid concentrate (L). Mean ± SEM. n = 10

Analysis of the survival proportions of Plasmodipur^®^ filtered and filter-retained ex vivo cultures at 48 h of incubation (Figs. 2, 3), detected a statistically significant difference in survival proportions between the filtered lipid (5/10) and the retained lipid (9/10) ex vivo cultures (*p* = 0.0255; one-tailed Chi square) (Fig. 3b), and a borderline significant difference between the filtered lipid (5/10) and retained serum (8/10) cultures (*p* = 0.0798; one-tailed Chi square), and filtered serum (6/10) and retained lipid (9/10) (*p* = 0.0607; one-tailed Chi square), suggesting that filtration was detrimental for the survival of ex vivo cultures independently of their media supplementation status. Similarly, no significant differences in the proportion of surviving cultures was detected between the retained serum (8/10) and retained lipid (9/10) ex vivo cultures (*p* = 0.2556; one-tailed Chi square) (Figs. 2 and 3b), indicating that filter-retained ex vivo cultures survived in higher proportions independently of their media supplementation status (Table 1). Taking together, this experiment demonstrates that filtration of leukocytes is not essential but appears deleterious for the survival of *P. vivax Aotus*-derived ex vivo cultures.

**Table 1:** Mean parasitaemia, developmental stages differential proportions, ring ratios and survival proportion of ten *Plasmodium vivax Aotus*-derived ex vivo cultures maintained with modified McCoy’s5A supplemented with 25% serum alone or 20% serum in combination with 5% chemically defined lipid concentrate

Time (hours)	Group	Mean parasitaemia					Ring Ratio	Survived/total
		%	Rings %	Trophozoites %	Schizonts %	Gametocytes %		
0	FS	0.4	54	31	14	1	1.17*	10/10
	FL	0.4	52	40	8	0	1.08*	10/10
	RS	0.7	39	45	15	1	0.64	10/10
	RL	0.7	40	46	13	1	0.67	10/10
24	FS	0.2	49	45	6	0	0.96	10/10
	FL	0.2	39	49	11	1	0.64	10/10
	RS	0.4	37	42	21	0	0.59	10/10
	RL	0.5	40	40	20	0	0.67	10/10
48	FS	0.1	13	41	46	0	0.15	6/10
	FL	0.1	28	72	0	0	0.39	5/10
	RS	0.1	40	27	33	0	0.67	8/10
	RL	0.2	35	35	30	0	0.54	9/10**

*p = 0.0292; one-tailed Chi square test

**p = 0.0255; one-tailed Chi square test

Ring ratio = rings/mature stages (trophozoites, schizonts and gametocytes)

*FS* filter serum, *FL* filtered lipid, *RS* retained serum, *RL* retained lipid

### Chemically defined lipid concentrate supplementation enhances the survival of filter retained *Aotus*-derived ex vivo cultures of *Plasmodium vivax*

**Fig. 4:**
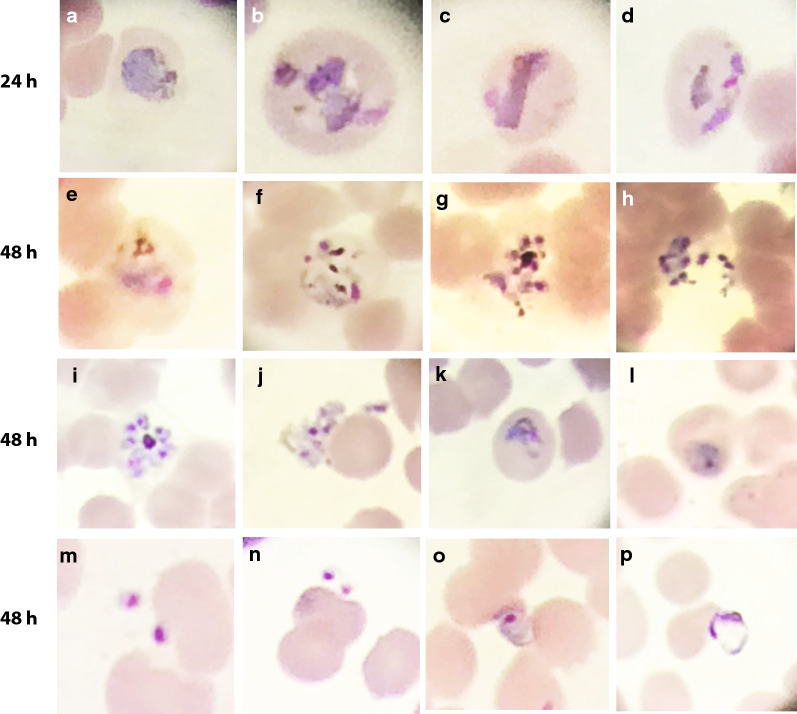
Representative light microscopic images of Giemsa-stained developmental stages of washed *Plasmodium vivax* in ex vivo cultures. Cultures from donor *Aotus* monkey 22033 were maintained with McCoy’s5A medium supplemented with 25% human serum alone. **a**–**d** Trophozoites in different stages of development. **e**, **f** Early schizont. **g**, **h** Ruptured schizonts. **i** Late schizont. **j** Ruptured schizont with merozoites attaching to an erythrocyte. **k** Trophozoite 48-h PI. **l** Re-invading early trophozoites. **m**, **n** Merozoite attached to an erythrocyte. **o**, **p** Extra-erythrocytic developing trophozoites (bizarre forms) indicative of failed invasion, notice swollen vacuole in p

**Fig. 5:**
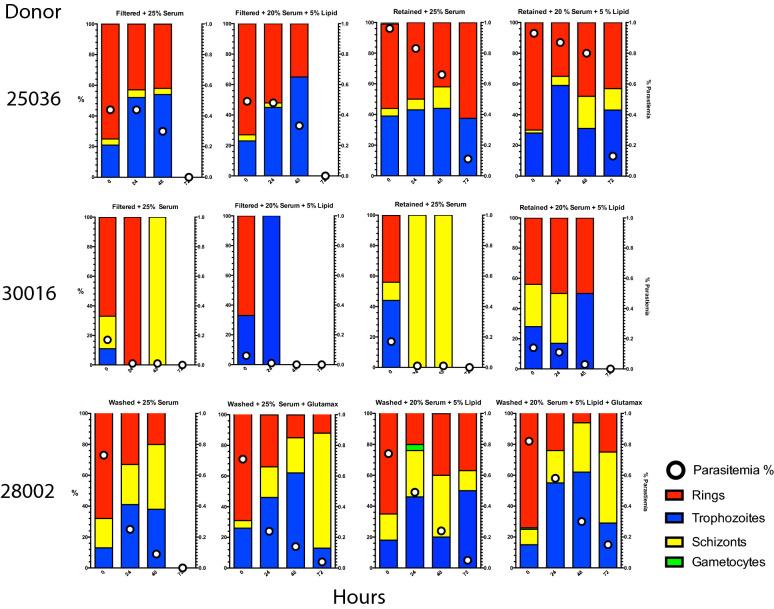
Mean parasitaemia and developmental stages differential proportions plots of *Plasmodium vivax* ex vivo cultures from selected individual *Aotus* donors. Mean of four technical replicates

To further characterize these findings, an analysis of the parasite maturation cycle of the ex vivo cultures was carried out, determining their ring ratios or proportion of rings to mature stages (ring ratio = rings/(trophozoites, schizonts and gametocytes) for each of the 10 *P. vivax Aotus* monkeys derived ex vivo cultures (Figs. 2, 3 and Table 1). This time, after 48 h of incubation, a statistically significant difference in the survival proportion of ex vivo cultures was observed in the retained lipid cultures compared to the filtered lipid (Fig. 3b) (*p* = 0.0255; one-tailed Chi square test), while there was no statistically significant difference between the retained serum and filtered serum cultures (*p* = 0.1646; one-tailed Chi square test). When the ring ratios and  % parasitaemia of the different treatment groups was analysed at time 0, 24 and 48 h of incubation, as shown in Fig. 3c, d, the parasites in the retained serum and retained lipid ex vivo cultures appeared cycling as evidenced by a higher relative proportion of rings (35–40%) (ring ratios: 0.67 and 0.54, respectively) observed in these two groups; this compared to only 13% in the filtered serum and 28% in the filtered lipid ex vivo cultures (ring ratios: 0.15 and 0.39, respectively) *(p* = 0.0004; one-tailed Chi square test) (Fig. 3b, c and Table 1), indicating that cycling in the retained cultures were maintained in a steady state as indicated by their constant rings ratios, which suffered little variation from baseline, compared to the filtered cultures that had a substantial drop by 48 h of incubation. Likewise, the mean parasitaemias in the retained lipid ex vivo cultures were significantly different compared to the filtered sera group at 24 h of incubation (*p* = 0.0218; unpaired two-tailed *t* test), but not at 48 h (Fig. 3d); suggesting that the parasites in the filtered sera cultures either had stalled (stop cycling) or failed at re-invading, as exemplified by the parasite bizarre forms compatible with failed re-invasions, observed attached but growing outside of RBCs at 48 h of incubation in washed only ex vivo cultures supplemented with 25% serum alone (Fig. 4, panels o-p**),** or were affected as shown in partially ruptured schizonts (Fig. 4, panels g, h, j). Strikingly, as shown in Fig. 5, by 72 h of incubation both the filtered sera and filtered lipid cultures of *Aotus* donors 25036 and 30016 were dead, but the retained serum and retained lipid cultures continued cycling, as evidenced by the higher proportion of schizonts observed in these groups (Fig. 5**)**. In contrast, the parasites in the washed ex vivo cultures derived from *Aotus* 28002 supplemented with 25% serum alone (Fig. 5), were all dead by 72 h of incubation. While, those supplemented with sera plus GlutaMax™, serum plus CDLC and serum plus CDLC and GlutaMax appeared cycling up to 72 h of incubation, suggesting a deleterious effect of filtration in *Aotus* donors 25036 and 30016 ex vivo *cultures*, but a protective effect of the CDLC supplement, even in the presence of low levels of WBCs. Nevertheless, it cannot be ruled out that the lower starting parasitaemias at time 0 h (< 0.2%) in the filtered cultures derived from *Aotus* 30016, composed of a higher proportion of rings (ring ratios: 1.17 and 1.08), when compared to the retained cultures (ring ratios: 0.64 and 0.67) (*p* = 0.0292; one-tailed Chi square test) (Fig. 3c**)**, might have played a role in the complete disappearance of the parasites by 72 h of incubation. Interesting to note is the low frequency of gametocytes present in *Aotus* blood at time of bleeding, when only 2/10 monkeys were positive for gametocytes (Additional file 1: Table S1), suggesting that the high passage *P. vivax* SAL-1 *Aotus*-adapted strain used in this study is a poor producer of gametocytes, as has been reported for squirrel monkeys (*Saimiri boliviensis*) infected with the Thai 561 strain of *P. vivax* [50].

Further analysis using parasitaemia fold change as an indicator of survival and cycling as shown in Fig. 3e, revealed that the retained lipid cultures maintained with McCoy’s5A medium supplemented with 20% serum plus CDLC, had the lowest mean negative fold parasitaemia change at 48 h of incubation (Mean ± SEM = -91 ± 79), compared to the filtered serum (Mean ± SEM = − 154 ± 71), filtered lipid (Mean ± SEM = − 161 ± 76), or retained serum cultures (Mean ± SEM = − 157 ± 96) (Additional file 1: Table S2), supporting a protective effect of CDLC supplementation.

### Chemically defined lipid concentrate plus GlutaMAX™ media supplementation improves the survival of washed *Plasmodium vivax Aotus*-derived ex vivo cultures

It is known that *Plasmodium* is incapable of de novo biosynthesis of fatty acids and cholesterol and must obtain it from the host [33], and that malaria parasites have a high demand for lipids to support their replication and enlarge their parasitophorous vacuole membrane (PVM) [38]. Hence, an experiment was design to test the effect of McCoy’s5A medium supplemented with 20% serum alone or in combination with 5% CDLC or GlutaMAX™ (a stabilized form of l-glutamine that prevents degradation and ammonia building in culture) or both, benchmarked against IMDM™ and AIM-V™ media; both that had been reported to be superior to McCoy’s5A [21, 28, 32] over parasitaemia levels of *P. vivax Aotus*-washed ex vivo cultures.

For this purpose, washed *P. vivax Aotus* iRBCs ex vivo cultures were maintained with McCoy’s5A modified, IMDM™ or AIM-V™ culture media as delineated in Fig. 1, determining their mean parasitaemia densities at 0, 24 and 48 h of incubation. After normalization of starting parasitaemias at time 0 h, by 24 h of incubation parasitaemia was significantly higher in the IMDM™ media group (*p *< 0.003; Mann–Whitney, two-tailed t-test), compared to McCoy’s5A medium supplemented with serum alone (reference group) or in combination with GlutaMAX™ (*p *< 0.004; Mann–Whitney, two-tailed t-test), but not with McCoy’s5A medium supplemented with serum plus CDLC alone or in combination with GlutaMAX™ (*p* = 0.1528; Mann–Whitney, two-tailed t-test), nor with the AIM-V™ culture medium group.

**Fig. 6:**
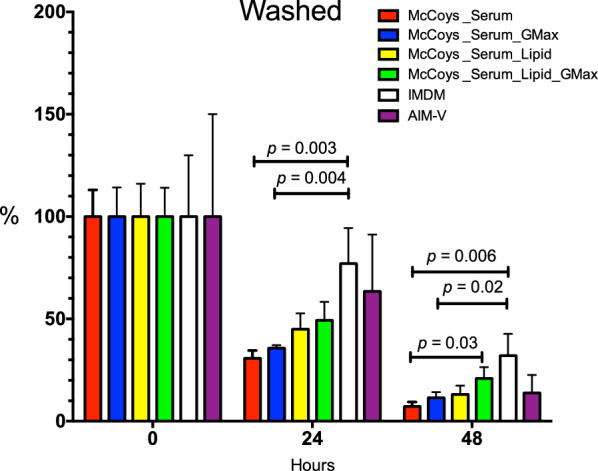
Mean parasitaemia plots of washed only *Plasmodium vivax Aotus* iRBCs ex vivo cultures. Cultures were maintained with McCoy’s5A medium supplemented with 20% serum alone or in combination with 5% CDLC and GlutaMAX™, IMDM™ or AIM-V™ media. Parasitaemias were normalized to time 0. Biological replicates (n) were maintained in cultures as follows: 25% serum only (n = 8); 25% serum + GlutaMAX™ (n = 7); 20% serum + CDLC (n = 7); 20% serum + CDLC + GlutaMAX™ (n = 7); IMDM^TM^ (n = 3); and AIM-V^TM^ (n = 3). Mean ± SEM. *p* = Mann–Whitney, two-tailed t-test. GMax = GlutaMAX™

By 48 h of incubation, parasitaemia was still significantly higher in the IMDM™ group, compared to McCoy’s5A supplemented with serum (reference group) (*p* = 0.006; Mann–Whitney, two-tailed t-test) or sera plus GlutaMAX™ (*p* = 0.02; Mann–Whitney, two-tailed t-test), but not with McCoy’s5A medium supplemented with serum plus CDLC and GlutaMAX™ (*p* = 0.3387; Mann–Whitney, two-tailed t-test), nor with the AIM-V™ medium (Fig. 6).

In general, an additive effect pattern of McCoy’s5A medium supplementation emerged as follows: serum < serum + GlutaMAX™ < serum + CDLC < serum + CDLC + GlutaMAX™ (Fig. 6), indicating the superiority of McCoy’s5A medium supplemented with serum in combination with CDLC and GlutaMAX™ over serum alone, and no significant difference in survival of parasites compared to IMDM™ and AIM-V™ media [27, 32], although substantial variation in parasitaemia densities was observed in both the IMDM™ and AIM-V™ media ex vivo culture groups.

### Human RBCs improved the survival of filtered Aotus *Plasmodium viv*ax-derived iRBCs ex vivo cultures

**Fig. 7:**
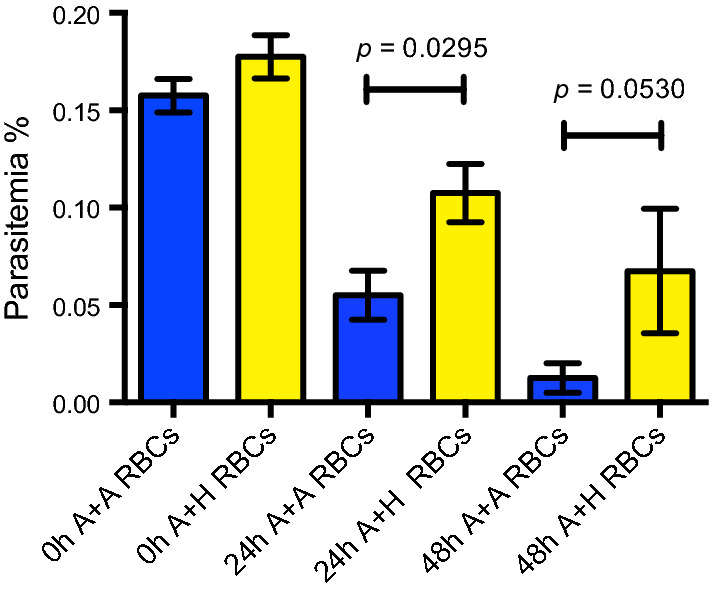
Mean parasitaemia of filtered *Plasmodium vivax Aotus*-derived iRBCs ex vivo cultures co-cultivated with *Aotus* or human malaria-naïve RBCs. Cultures were maintained with McCoy’s5A medium supplemented with 25% human serum or co-cultivated with *Aotus* (A) or human (H) malaria-naïve RBCs. Mean ± SEM of four technical replicates. *p* = Statistical significance Mann–Whitney U non-parametric one-tail t-test. Donor *Aotus* MN29038: parasitaemia density = 142.1 × 10^3^ × μL; stage differential proportions = rings 83%, trophozoites 27%, schizonts 0%; post-inoculation day 15

**Fig. 8:**
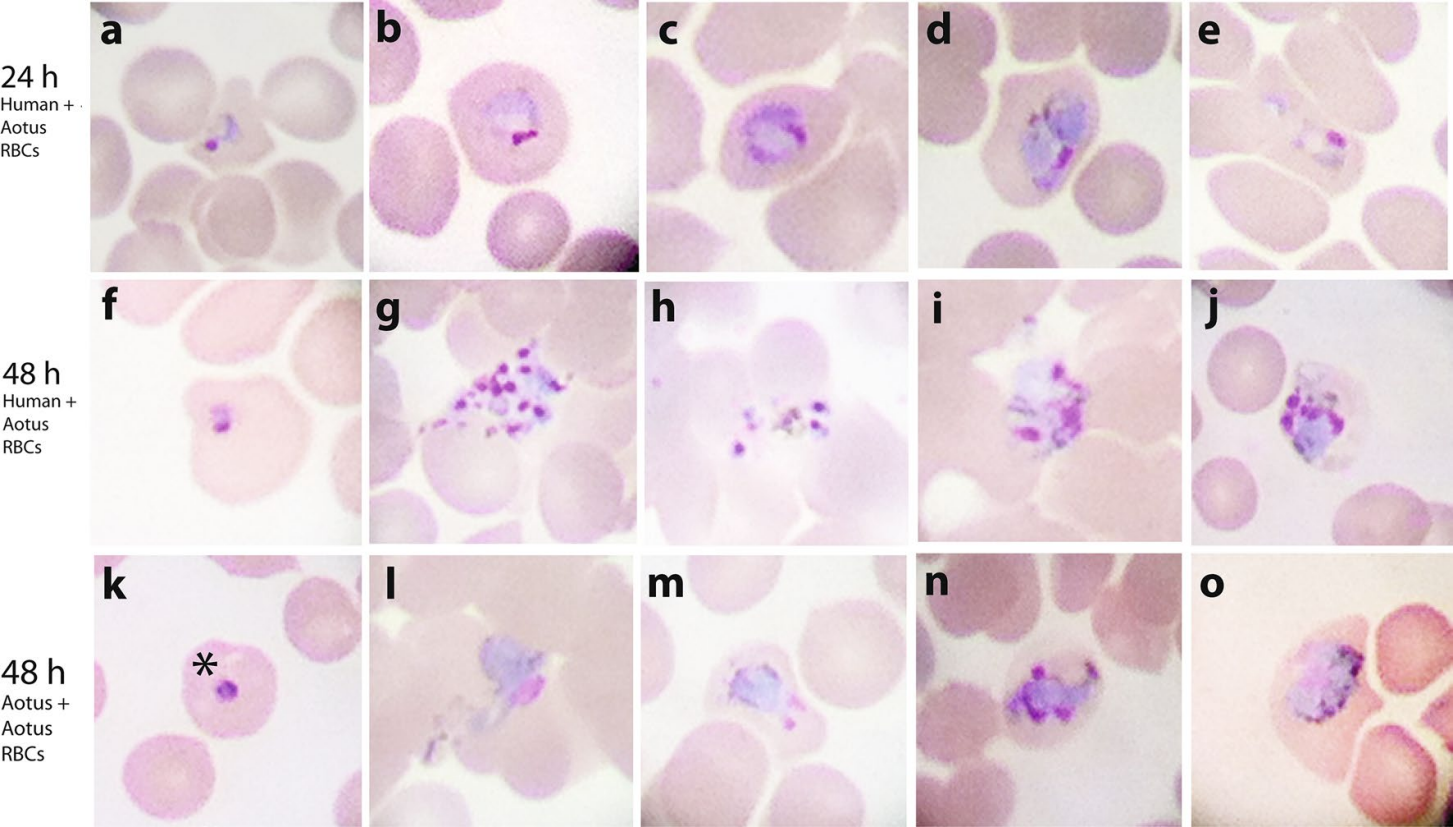
Selected light microscopic images of Giemsa-stained developmental stages of co-cultivated *Aotus*-derived *Plasmodium vivax* ex vivo cultures. Cultures were co-cultivated with *Aotus* or malaria-naïve hRBCs. **a**–**e** Images of 24-h ex vivo cultures co-cultivated with malaria-naive hRBCs supplemented with 25% sera showing early and late trophozoites. **f**–**j** Images of 48-h ex vivo cultured co-cultivated with malaria-naive hRBCs, showing rings, schizonts and bursted schizonts. **k**–**o** Images of 48-h ex vivo culture co-cultivated with malaria-naive *Aotus* RBCs only, showing rings, trophozoites and schizonts. * show early trophozoite (ring)

Next, to test the effect of co-cultivation with hRBCs on the survival of *Aotus*-derived *P. vivax* ex vivo cultures, *Aotus* iRBCs were co-cultivated with malaria-naïve *Aotus* or hRBCs in a 50:50 proportion at 5% HTC concentration, and maintained with McCoy’s5A medium supplemented with 25% sera. This time, by 24 h of incubation, parasitaemia densities in the hRBCs co-cultivated ex vivo cultures were significantly higher than in the *Aotus* blood-only supplemented cultures (*p* < 0.0295; Mann–Whitney U non-parametric one-tail test) (Fig. 7), with re-invasions observed in both cultures at 48 h of incubation (Fig. 8), though there was only a borderline statistically significant difference in parasitaemia densities between groups (*p* = 0.0530; Mann–Whitney U non-parametric one-tail test), suggesting that co-cultivation with hRBCs improved the survival of *P. viv*ax *Aotus*-derived iRBCs ex vivo cultures.

## Discussion

The serendipitous finding that a *P. vivax*-positive clinical sample inoculated into an *Aotus* monkey remained viable after being left at room temperature for three days, prompted the hypothesis of this study, that filtration of WBCs was not essential for the survival of *P. vivax* ex vivo cultures, but that leukocytes may play a role at maintaining a homeostatic milieu mimicking whole blood conditions ex vivo. In fact, in 1912 Bass and Johns [39] during their *“*tertian malaria*”* ex vivo culture attempts, working at Ancon Hospital in the Panama Canal Zone, reported malaria parasites developing at the top of a column of defibrinated whole blood without removal of leukocytes, even after being left at room temperature for 4 days, with rapid growth obtained at temperatures of 40 to 41 °C under strict anaerobic conditions [39].

This study demonstrates that Plasmodipur^®^ filter-retained *P. vivax Aotus*-derived iRBCs ex vivo cultures maintained with McCoy’s5A medium supplemented with 20% serum plus 5% CDLC, had superior survival rates compared to filtered ex vivo cultures, suggesting that the presence of leukocytes in the retained cultures was not a limiting factor for the improved survival observed. However, it cannot rule out that other factors, such as a the low recovery rate of schizonts and gametocytes found after Plasmodipur^®^ filtration as it has been reported in other studies [42]; as well as factors such as physical forces (pressure) exerted over the parasites during filtration, which might have resulted deleterious to iRBCs (lysis); or a reduce invasion efficiency (fitness), as evidenced by the observation of parasites growing attached outside of erythrocytes (bizarre forms), as it has been observed by others [51]; might have all or in part contributed to the low starting and final parasitaemias observed in the filtered ex vivo cultures of this study.

McCoy’s5A medium supplemented with cholesterol concentrates (750 mg/L) alone or in combinations with several other supplements, has been used in *P. vivax* ex vivo cultures without significant improvements in parasite growth [31]. This study demonstrates that McCoy’s5A medium supplemented with serum plus CDLC and GlutaMAX™ (McCoy’s5A complete), improves the survival of washed *P. vivax Aotus*-derived ex vivo cultures, suggesting that during replication, *Aotus*-derived *P. vivax* parasites are in high demand for lipids, amino acids and other building blocks, such as l-glutamine [52], as it has been reported for *P. falciparum* and other *Plasmodium* species during replication in the liver [33, 38]. Moreover, data analysis could not detect significantly statistical differences in parasitaemia densities by 48 h of incubation when McCoy’s5A complete was benchmarked against IMDM™; this that had been claimed to be superior through a single maturation cycle of the parasite [28, 32], nor to AIM-V™ used in the long-term in vitro culture of *P. vivax* in *Saimiri boliviensis* blood [27], revealing a pattern of McCoy’s5A medium supplementation for *P. vivax Aotus*-derived ex vivo cultures as follows: serum < serum + GlutaMAX™ < serum + CDLC < serum + CDLC + GlutaMAX™; confirming a key role of the CDLC supplementation and the additive effect of serum plus CDLC plus GlutaMAX™ on the enhanced survival observed in the *Aotus*-derived *P. vivax* ex vivo cultures.

At this point it is important to note that during replication, *Plasmodium* parasites are in high demand for lipids and cholesterol that must be obtained from the host erythrocytes [33]. Acquisition of host lipids is fundamental for the intra-erythrocytic state, when the parasite is only capable of storing lipids within its parasitophorous vacuole [53]. Cholesterol and lipid content of the infected erythrocyte increases during replication, and is a reflection of the contributions of the erythrocyte, the parasite and blood plasma; all of which show alterations during infection [21, 33, 54, 55]. For instance, total cholesterol is decreased in rhesus monkeys infected with *Plasmodium coatneyi* and vary between uninfected and infected erythrocytes of ducks, macaques and rats with their respective malarial parasites [33]. In the *P. vivax Aotus* ex vivo co-cultivation experiment, a significant difference in survival rates of ex vivo cultures were evident in the malaria-naïve human erythrocytes supplemented cultures. As such, it could be speculated that the sources of lipids available to the parasite during replication in these co-cultures, apart from the added serum and the *Aotus* monkey erythrocytes, may had been provided by the malaria-naïve human erythrocytes added to the cultures in a 50:50 proportion. If such was the case, it could indicate that human erythrocytes played an important role as a source of lipids and other important nutrients to the parasites. This observation warrants further investigation.

As Bass and Johns pointed out during their ex vivo culture experiments [39], that tertiary malaria parasites seemed more “serviceable” for cultivation after 1–2 h of the patient having ingested a complete meal, but could not grow in blood after fasting, even in the presence of dextrose, points out to the importance of lipids (enriched in lymphatic chyle during the post-prandial stage of digestion) in malaria infection, as has also been observed in fasting mice refractory to malaria [56]. Importantly to note is that in these series of experiments, *P. vivax*-infected blood was collected from fasting *Aotus* monkeys. Future experiments will need to consider bleeding of *P. vivax*-infected blood donors soon after feeding, to account for this seemingly important variable.

This study has some limitations: For instance, the proportion of WBCs in the cultures present at time 0, was not determined, nor the types of cells (*Aotus* or human), that were invaded by the parasite in the co-culture experiment. Furthermore, experimental repetitions of some ex vivo cultures were limited by the availability of *P. vivax*-infected *Aotus* monkey blood donors. Future experiments to account for the proportion of WBCs present in culture at time 0, determine the side-by-side longitudinal changes in composition between *Aotus* and human erythrocytes in culture, as well as differentiate between invaded *Aotus* and human iRBCs using flow cytometry, will follow.

## Conclusions

Altogether, this study demonstrates that WBCs filtration is not essential for the survival of *P. vivax Aotus*-derived ex vivo cultures. It also demonstrates that McCoy’s5A medium supplemented with CDLC improves the survival of retained ex vivo cultures irrespective of WBCs presence. Moreover, it demonstrates that McCoy’s5A medium supplemented with serum plus CDLC and GlutaMAX™ improves the survival of *Aotus*-derived *P. vivax* ex vivo cultures, and was not significantly different compare to IMDM or AIM-V culture media. Finally, the study shows that co-cultivation with hRBCs enhances the survival of *P. vivax Aotus*-derived ex vivo cultures by a yet undetermined mechanism. The results of the study contribute further to the optimization of seeding material for long-term in vitro culture attempts using Aotus derived *P. vivax*, underscoring the importance of filtration and culture media supplementation on the survival of *P. vivax Aotus*-derived ex vivo cultures.

## Supplementary information

**Additional file 1: Table S1.** Individual P. vivax SAL-1 infected Aotus monkeys parasitemias of filtered or retained iRBCs ex-vivo cultures incubated with McCoy’s5A media supplemented with serum and lipiid concentrate alone or in combination.

**Additional file 2: Figure S1.**

## Data Availability

All data generated or analysed during this study are included in this published article and its supplementary information files.

## Abbreviations

ACD: Acid Citrate Dextrose
CDLC: Chemically Defined Lipid Concentrate
GMIHS: Gorgas Memorial Institute of Health Studies
HCT: Haematocrit
IFA: Immunofluorescence Assay
IMDM™: Iscove’s Modified Dulbecco’s Medium
hRBCs: Human Red Blood Cells
iRBCs: Infected Red Blood Cells
PBS: Phosphate Buffer Saline
PVM: Parasitophorous Vacuole Membrane
RBCs: Red Blood Cells
WBCs: White Blood Cells
