# Supplementary figures and images for: On the survival of 48 h Plasmodium vivax Aotus monkey-derived ex vivo cultures: the role of leucocytes filtration and chemically defined lipid concentrate media supplementation
Source: Malar J. 2020 Aug 3;19:278. doi: 10.1186/s12936-020-03348-9 (PMC7398384; doi:10.1186/s12936-020-03348-9)

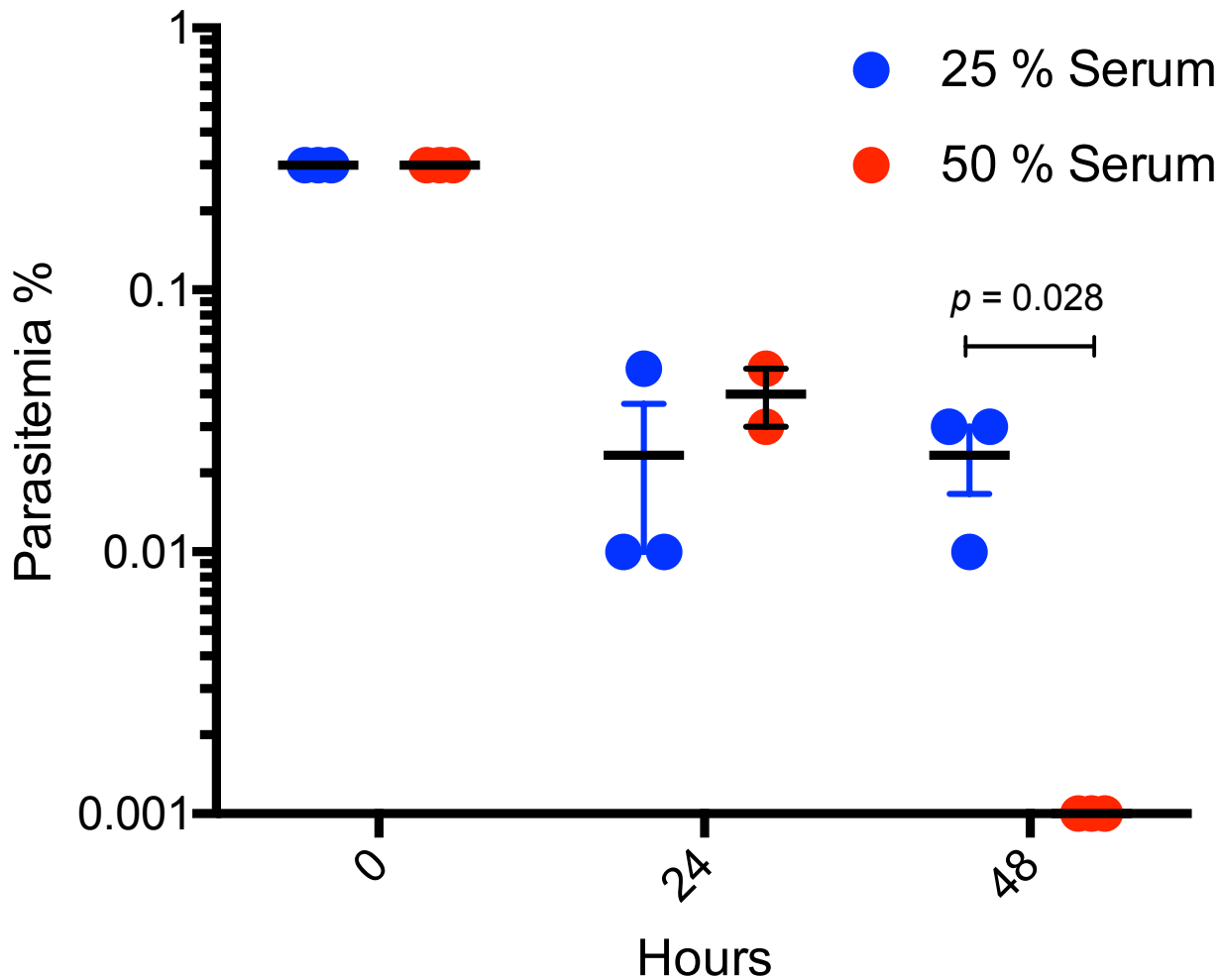

Supplement: Supplementary file 2 — Additional file 2: Figure S1. [file 12936_2020_3348_MOESM2_ESM.pdf]
